# Analgesic Efficacy of Preemptive Transversus Abdominis Plane Block in Patients Undergoing Laparoscopic Colorectal Cancer Surgery

**DOI:** 10.3390/jcm9051577

**Published:** 2020-05-22

**Authors:** Kwan Young Hong, Duk Kyung Kim, Hue Jung Park, Woo Seog Sim, Won Gook Wi, Woo Yong Lee, Hee Cheol Kim, Jin Young Lee

**Affiliations:** 1 Department of Anesthesiology and Pain Medicine, Samsung Medical Center, School of Medicine, Sungkyunkwan University, Seoul 06351, Korea; kwanyoung.hong@samsung.com (K.Y.H.); dk68.kim@samsung.com (D.K.K.); wooseog.sim@samsung.com (W.S.S.); wongook.wi@samsung.com (W.G.W.); 2 Department of Anesthesiology and Pain Medicine, Seoul St. Mary’s Hospital, College of Medicine, The Catholic University of Korea, Seoul 06591, Korea; huejung@catholic.ac.kr; 3 Department of Surgery, Samsung Medical Center, School of Medicine, Sungkyunkwan University, Seoul 06351, Korea; wooyong123.lee@samsung.com (W.Y.L.); hc111.kim@samsung.com (H.C.K.)

**Keywords:** acute, colorectal cancer, laparoscopic, postoperative pain, TAP block

## Abstract

Despite rapid advancements in laparoscopic surgical devices and techniques, pain remains a significant issue. We examined the efficacy of preemptive transversus abdominis plane (TAP) block for acute postoperative pain in patients undergoing laparoscopic colorectal cancer surgery. We retrospectively analyzed 153 patients who underwent laparoscopic colorectal cancer surgery with or without TAP block; among them, 142 were allocated to the TAP or non-TAP group. We performed between-group comparisons of demographic, clinical, and anesthetic data and pain scores at a postoperative anesthesia care unit (PACU) and at postoperative days 1, 3, and 5. There were no significant between-group differences in demographic and clinical characteristics. The mean arterial pressure, heart rate, and minimum alveolar concentration (MAC) were significantly lower in the TAP group at the start and end of surgery. The post-extubation bispectral index was significantly higher in the TAP group. There were no significant between-group differences in the pain scores and opioid consumption at the PACU or at postoperative days 1, 3, and 5, or in the time to pass flatus, the hospital stay length, and postoperative complications. Preemptive TAP block showed an intraoperative, but not postoperative, analgesic effect, characterized by a low mean arterial pressure, heart rate, and MAC.

## 1. Introduction

Despite rapid advancements in laparoscopic surgical devices and techniques, postoperative pain remains a significant clinical issue [1,2,3]. Postoperative pain is insufficiently treated in 30–40% of patients; moreover, it affects postoperative recovery, discharge, and quality of life [2,4,5,6,7]. Since surgical incisions cause peripheral and central sensitizations, preemptive analgesic management is essential for attenuating nociception and pain sensitization processes [8,9,10]. As an element of multimodal analgesia for enhanced recovery after surgery (ERAS), transversus abdominis plane (TAP) block has varying efficacy degrees [10,11,12]. TAP block is a regional technique for analgesic effects on the anterolateral abdominal wall and parietal peritoneum that cover the anterior division of the T6 to the L1 spinal nerve, which runs into the plane between the internal oblique and transversus abdominis muscles [13]. TAP block improves postoperative pain in several abdominal surgeries, including colectomy, nephrectomy, hysterectomy, cholecystectomy, and hernia repair [8,11,13,14,15]. Damadi and colleagues [16] reported that the combined use of TAP block and the ERAS pathway reduced the use of postoperative narcotics, time to ambulation and bowel function, and hospital stay length in laparoscopic colorectal surgeries. However, various TAP block protocols (with varying local anesthetic volume and concentration, as well as adjuvant agent use), approach methods (lateral, posterior, or subcostal), injection times, continuous infusion uses, and surgery types have varying effects [8,14,16,17]. TAP block has been reported to predominantly and acutely inhibit somatic pain [18,19]. Sun and colleagues [18] reported that TAP block using 0.375–0.5% ropivacaine reduced postoperative pain for 2 h and reduced the incidence of nausea and vomiting in the 24 h follow-up period. However, increasing the local anesthetic concentration and/or volume is a significant factor for systemic toxicity given the rich venous and arterial network within the transversus abdominis plane [19]. A recent study reported that TAP block with liposomal bupivacaine decreased postoperative pain within a 3 day follow up after open and laparoscopic colorectal surgery [20]. However, there remain issues regarding cost-effectiveness and Food and Drug Administration approval in each country [21]. There has been no study on TAP block with adjuvant hydromorphone with respect to analgesic prolongation. We aimed to evaluate the analgesic efficacy of preemptive TAP block with ropivacaine and hydromorphone in patients undergoing laparoscopic colorectal cancer surgery.

## 2. Materials and Methods

### 2.1. Patients

We retrospectively reviewed the electronic medical records of patients who underwent elective laparoscopic colorectal surgery between October 2019 and February 2020 at a single center. The age of the patients ranged from 28 to 86 years. All patients had colon or rectum tumors and had undergone laparoscopic colectomy or anterior resection [22]. We used the following exclusion criteria: emergent surgery, metastatic colorectal lesions, conversion to open surgery, a lack of follow-up data, needing postoperative intensive care unit management, an inability to express pain severity, and patients taking analgesics (acetaminophen, a non-steroidal anti-inflammatory agent, tramadol, or a glucocorticoid) during anesthesia [22]. This study was approved by our departmental ethics committee (ref: SMC 2020–04–065) and registered with CRIS (Clinical Research Information Service of the Korea National Institute of Health, http://cris.nih.go.kr/cris/index.jsp, ref: KCT0004920).

### 2.2. TAP Block and Anesthesia

There was no preoperative premedication. Upon admission to the operating room, standard monitoring (IntelliVue MP70, Philips Healthcare, Best, The Netherlands) was performed, including oxygen saturation determination, electrocardiography, end-tidal carbon dioxide determination, pulse oximetry, bispectral index (BIS) determination, and non-invasive blood pressure determination. The patients were placed in the supine position, exposing the costal margin and iliac crest. After aseptic preparation, a 12 MHz linear transducer was placed above the iliac crest. Subsequently, 2 mL of 1% lidocaine was infiltrated at the location of the rectus abdominis muscle tapering in an anterior axillary line with clear visualization of the three abdominal wall muscle layers. Subsequently, a 23-gauge needle (Tae-Chang Industrial Co., Seoul, Korea) was inserted in the plane between the internal oblique and transversus abdominis muscles. After confirming an appropriate needle position, a 20 mL mixture of 0.15% ropivacaine and 0.5 mg of hydromorphone was injected. The horizontal spread of injectate was visualized with ultrasound. TAP block was performed on the bilateral side. All blocks were performed by the same experienced anesthesiologist (J.Y.L.). After TAP block completion, anesthesia was induced using an intravenous injection of 40 mg of 2% lidocaine, 2 mg/kg of 2% propofol, 0.5–1 µg/kg of fentanyl, 0.05 mg/kg of midazolam, and 0.6–0.8 mg/kg of rocuronium. Endotracheal intubation was performed using a Macintosh laryngoscope after mask ventilation for approximately 3–5 min and the loss of all four twitches elicited by train-of-four ulnar nerve stimulation. After tracheal intubation, anesthesia was maintained with 1.5–3.0 vol% sevoflurane and a bolus injection of 0.5–1 µg/kg of fentanyl for maintaining hemodynamic parameters within 20% of the baseline values and the BIS at 40–60. The lungs were ventilated using 50% oxygen with air with adjustment for maintaining an end-tidal carbon dioxide level of 30–40 mmHg. All surgeries were performed by one of six specialized colorectal surgeons following techniques similar to those for colorectal cancer [23]. Approximately 20 min before the end of surgery, the patients were connected to an intravenous patient-controlled analgesia pump (Automed3200^®^, Ace Medical, Seoul, Korea), which delivered 20 µg/kg of fentanyl in normal saline (100 mL) at a basal infusion rate of 0.5 mL/h and a 1 mL bolus. The lockout interval was 15 min. At the end of surgery, the patients received an intravenous injection of 0.4 mg/kg of sugammadex. After extubation, the patients were moved to the postoperative anesthesia care unit (PACU). Furthermore, they received an intravenous bolus of 0.5 µg/kg of fentanyl when the numeric rating scale (NRS, 0 = no pain to 10 = absolutely intolerable pain) scores were >3. Postoperative opioids at the general ward included intravenous fentanyl, pethidine, or hydromorphone, as well as oral medications, including oxycodone and tapentadol. Opioid consumption was recorded by conversion to fentanyl units throughout the postoperative days (PODs) 1, 3, and 5 [23]. Pain scores were recorded at PACU arrival and PACU discharge, as well as at PODs 1, 3, and 5.

### 2.3. Statistical Analysis

All statistical analyses were performed using SAS 9.4 (SAS Institute, Cary, NC, USA). Data were expressed as the mean ± standard deviation (SD) or number (proportion), as appropriate. Between-group comparisons of demographic, clinical, and anesthetic characteristics were performed using a chi-square test, t-test, or Fisher’s exact test. We extracted the mean arterial pressure, heart rate, and BIS before intubation (T1), at the start of surgery (T2), at the end of surgery (T3), and after extubation (T4). The minimum alveolar concentration (MAC) of sevoflurane was determined at T2 and T3 and compared using the t-test or Wilcoxon rank-sum test. We compared pain scores at PACU arrival and discharge, as well as PODs 1, 3, and 5, with rest and movement. Moreover, we compared opioid consumption at anesthesia, the PACU, and at PODs 1, 3, and 5 using the Wilcoxon rank sum test. The postoperative complications were compared using the chi-square test. The statistical significance level was set at *p* < 0.05.

## 3. Results

Among the 153 patients assessed for eligibility, 11 were excluded for having insufficient medical records. Therefore, we analyzed data from 142 patients. The patients were categorized into two groups based on whether they had undergone TAP block (TAP group, *n* = 70) or not (non-TAP group, *n* = 72). Table 1 summarizes the demographic and clinical characteristics. There were no significant between-group differences in the age, sex, body mass index, American Society of Anesthesiologists (ASA) status, diagnosis, preoperative chemoradiotherapy (CRT), surgery type, pathological stage, maximum tumor size >4 cm, ileostomy, operation and anesthesia times, and intraoperative fentanyl dose (Table 1). Intraoperatively, the TAP group had a significantly lower mean arterial pressure at T2 (*p* = 0.004) and T3 (*p* = 0.003), and heart rate at T2 (*p* < 0.000) and T3 (*p* = 0.002). The TAP group had a significantly lower MAC of sevoflurane at T2 (*p* = 0.002) and T3 (*p* = 0.020), and a significantly higher BIS at T4 (*p* = 0.038) (Table 2). There were no significant differences in the postoperative pain scores across the time points from PCAU to POD 5 (Table 3). Postoperatively, there was no between-group difference in fentanyl consumption across the time points or in the time to pass flatus and hospital stay length (Table 4). Moreover, there was no between-group difference in the postoperative incidence of nausea/vomiting, itching, anastomotic leak, anastomotic hemorrhage, infection of the incision, and ileus (Table 5).

## 4. Discussion

This study aimed to evaluate whether preemptive TAP block with ropivacaine and hydromorphone exerts an analgesic effect on acute postoperative pain in patients undergoing laparoscopic colorectal cancer surgery. We observed an intraoperative analgesic effect characterized by a decreased mean arterial pressure, heart rate, and MAC of sevoflurane. TAP block did not reduce postoperative pain scores or opioid consumption; accordingly, there was no between-group difference in the incidence of postoperative nausea/vomiting and itching. We suspect that laparoscopic colorectal surgical pain may be predominantly visceral or neuropathic pain rather than somatic pain. Recent colorectal surgery has seen technical improvement for invasiveness reduction via smaller incisions, which could be associated with reduced somatic pain [3]. However, TAP block alone cannot alleviate the visceral pain caused by bowel manipulation and the intraoperative damage to nerves innervating visceral structures. Therefore, TAP block can be used as part of multimodal analgesia under general anesthesia; moreover, we observed that it was insufficient for analgesia during the postoperative period, which could be attributed to the visceral pain nature of colorectal surgery. This is consistent with previous reports that the TAP block did not cover visceral pain [10,19,24,25].

ERAS protocols in colorectal cancer resection are designed to reduce the hospital stay length, readmission rate, and postoperative complications, thereby improving patient outcomes [26,27]. ERAS protocols include 26 components (three preadmission, eight preoperative, six intraoperative, and nine postoperative) [26,27]. ERAS guidelines recommend a multimodal approach to pain control, which is based on the combined use of opioid and opioid-sparing analgesia involving the concurrent use of multiple non-opioid analgesics and regional analgesia, including neuraxial (epidural analgesia and spinal morphine) and peripheral (TAP, paravertebral, brachial plexus, or sciatic/femoral blocks, or wound infiltration) block [10]. The abdominal wall is blocked through local anesthetic injection within musculofascial planes by anesthetizing multiple small nerves or plexuses [19]. This can involve TAP, ilioinguinal–iliohypogastric, rectus sheath, and transversalis fascia plane block [19]. TAP block in colorectal surgery covers the six lower thoracic spinal nerves (T7–T12) to relieve surgical traumatic pain at the abdominal wall. Lateral TAP block generally provides analgesia at the T10–T12 dermatome with a lesser effect at the T9 and L1 dermatome [19,28]. Postoperative pain after colorectal surgery involves a combination of somatic, visceral, and neuropathic pain. Moreover, since the pelvis is narrow, there is an increased risk of intraoperative damage to the autonomic or somatic pelvic nerve plexus [23,29,30]. Rashid and colleagues [31] reported that there was no difference in the analgesic efficacy of bilateral lateral TAP block and the local anesthetic infiltration of the port and extraction site within 48 postoperative hours. This indicates that TAP block efficacy involves somatic block at the abdominal wall wound. Smith and colleagues [25] reported that bilateral lateral TAP block with ropivacaine did not improve postoperative pain within 72 postoperative hours. The approximate ropivacaine blockade duration in TAP block is 4–8 h [32]. We referred to the mean operation time (100–177 min) from previous reports and added hydromorphone in anticipation of analgesic prolongation [3,22,23,25]. Hydromorphone is a semi-synthetic morphine derivative that is five times more potent than morphine [7]. It has moderate hydrophilicity, which allows faster crossing of the blood–brain barrier, as well as a faster onset and modest duration of action [33]. Neuraxial hydromorphone in brachial plexus block with ropivacaine has been reported to induce prolonged sensory and motor block; however, there has been no study on the adjuvant use of hydromorphone in TAP block [33]. Our findings indicate that adding hydromorphone in lateral TAP block does not affect postoperative pain. The colorectum is innervated by lumbar splanchnic and pelvic nerves, as well as afferent nerves from the thoracolumbar and lumbosacral dorsal root ganglia [34] Visceral pain from the colorectum has psychophysical characteristics and is caused by mechanical distension, stretch, or bowel ischemia rather than noxious stimuli from skin incision [34]. Therefore, although TAP block with hydromorphone significantly stabilizes intraoperative hemodynamics under general anesthesia, it cannot attenuate postoperative lumbar splanchnic and pelvic nerve irritation. There is a need for future studies to determine novel modalities for the ERAS pathway that cover visceral pain.

This study had several limitations. First, we could not exclude the systemic analgesic effect of ropivacaine and hydromorphone in TAP block since we did not determine their plasma concentrations. Second, we did not individually assess somatic (incisional) and visceral (deep abdominal) pain characteristics; therefore, we could not exactly determine the analgesic effect of TAP block on each pain type. Third, we did not include a sham group for comparing the efficacy of TAP block without hydromorphone. Fourth, we excluded patients who intraoperatively received non-opioid analgesics, including acetaminophen, a non-steroidal anti-inflammatory agent, tramadol, or a glucocorticoid, since it is difficult to compare different forms and combinations of analgesics that may affect postoperative pain. A previous study reported that multi-model pain concepts of ERAS could not decrease morphine consumption because of the unstandardized opioid-sparing strategies in colorectal surgery [35]. Fifth, the sample size of our study was small.

## 5. Conclusions

In conclusion, preemptive lateral TAP block with ropivacaine and hydromorphone showed an intraoperative, but not postoperative, analgesic effect characterized by a low mean arterial pressure, heart rate, and MAC. There is a need for further prospective randomized studies including sham blocks with larger sample sizes to evaluate analgesic prolongation through differing concentrations of local anesthetics as part of the TAP block, as well as combined approaches that use different regional anesthesia techniques and other adjuvants.

## Figures and Tables

**Table 1:** Demographic and clinical characteristics.

	All Patients	Non-TAP Group	TAP Group	*p*-Value
	(*n* = 142)	(*n* = 72)	(*n* = 70)	
Age (y)	61.2 ± 11.5	60.8 ± 11.8	61.7 ± 11.3	0.645
Sex (M/F)	78/64	42/30	36/34	0.408
Body mass index (kg/m^2^)	24.4 ± 3.2	24.2 ± 3.2	24.7 ± 3.2	0.333
ASA status: I/II/III	71/61/10	41/26/5	30/35/5	0.223
Diagnosis				0.569
Colon cancer	90 (63.4%)	44 (61.1%)	46 (65.7%)	
Rectal cancer	52 (36.6%)	28 (38.9%)	24 (34.3%)	
Preoperative CRT (yes/no)	5/137	2/70	3/67	0.679
Surgery type				0.138
Colectomy	33 (23.2%)	13 (18.1%)	20 (28.6%)	
Anterior resection	109 (76.8%)	59 (81.9%)	50 (71.4%)	
Pathological stage				0.706
0/1/2/3/4	20/13/28/28/53	13/7/13/14/25	7/6/15/14/28	
Maximum tumor size >4 cm	40 (28.2%)	21 (29.2%)	19 (27.1%)	0.789
Ileostomy	5 (3.5%)	2 (2.8%)	3 (4.3%)	0.679
Operation time (min)	139.9 ± 52.2	136.4 ± 53.3	143.5 ± 51.2	0.345
Anesthesia time (min)	185.2 ± 57.8	182.3 ± 60.6	188.2 ± 55.1	0.355
Preoperative pain (NRS)	0.0 ± 0.0	0.0 ± 0.0	0.0 ± 0.0	1.000
Rocuronium dose (mg)	76.6 ± 18.2	77.9 ± 17.8	247.6 ± 65.5	0.331
Sugammadex dose (mg)	251.3 ± 54.9	255.0 ± 42.3	247.6 ± 65.5	0.687
Intraoperative fentanyl (µg)	75.9 ± 32.3	73.6 ± 31.9	78.2 ± 32.7	0.341

All data are presented as the mean ± SD or number (%) of patients. ASA: American Society of Anesthesiologists, CRT: chemoradiotherapy, NRS: numeric rating scale; Non-TAP group: patients who did not undergo TAP block, TAP group: patients who underwent TAP block; *p*-value < 0.05 was considered statistically significant.

**Table 2:** Intraoperative vital signs, minimum alveolar concentration, and bispectral index over time.

	All Patients	Non-TAP Group	TAP Group	*p*-Value
	(*n* = 142)	(*n* = 72)	(*n* = 70)	
Mean arterial pressure				
T1	92.5 ± 10.6	92.8 ± 9.6	92.2 ± 11.5	0.752
T2	85.6 ± 17.8	89.6 ± 17.8	81.6 ± 16.9 *	0.004
T3	84.0 ± 13.0	87.1 ± 13.7	80.7 ± 12.2 *	0.003
T4	95.4 ± 13.9	95.9 ± 12.6	94.9 ± 15.2	0.651
Heart rate				
T1	73.5 ± 12.5	72.5 ± 11.2	74.5 ± 13.8	0.399
T2	76.1 ± 14.8	80.4 ± 14.5	71.7 ± 14.0 *	<0.000
T3	66.9 ± 12.2	70.1 ± 13.1	63.7 ± 10.4 *	0.002
T4	81.2 ± 11.5	82.0 ± 12.3	80.3 ± 10.6	0.369
MAC of sevoflurane				
T2	1.3 ± 0.4	1.4 ± 0.4	1.2 ± 0.3 *	0.002
T3	0.6 ± 0.4	0.6 ± 0.3	0.5 ± 0.4 *	0.020
Bispectral index				
T1	93.0 ± 6.5	93.3 ± 5.0	92.6 ± 7.8	0.897
T2	40.4 ± 9.1	39.3 ± 8.9	41.4 ± 9.2	0.085
T3	53.5 ± 9.5	53.3 ± 10.6	53.8 ± 8.2	0.476
T4	83.9 ± 7.3	82.6 ± 7.2	85.2 ± 7.2 *	0.038

All data are presented as the mean ± SD. MAC: minimum alveolar concentration, T1: before intubation, T2: start of surgery, T3: end of surgery, T4: after extubation; Non-TAP group: patients who did not undergo TAP block, TAP group: patients who underwent TAP block; * *p*-value < 0.05 was considered statistically significant.

**Table 3:** Postoperative pain score.

Postoperative Pain (NRS)	All Patients	Non-TAP Group	TAP Group	*p*-Value
	(*n* = 142)	(*n* = 72)	(*n* = 70)	
PACU				
At admission	6.2 ± 1.9	6.5 ± 1.9	5.9 ± 1.9	0.134
At discharge	2.6 ± 0.9	2.7 ± 0.8	2.6 ± 1.0	0.455
POD 1				
Rest	2.9 ± 0.3	2.9 ± 0.3	2.9 ± 0.3	>0.999
Movement	4.6 ± 1.9	4.5 ± 1.8	4.8 ± 1.9	>0.999
POD 3				
Rest	2.9 ± 0.4	2.9 ± 0.4	2.9 ± 0.3	>0.999
Movement	3.5 ± 1.3	3.7 ± 1.5	3.3 ± 1.1	0.297
POD 5				
Rest	2.9 ± 0.4	2.9 ± 0.5	3.0 ± 0.7	>0.999
Movement	3.0 ± 0.7	3.0 ± 0.7	3.0 ± 0.7	>0.999

All data are presented as the mean ± SD. NRS: numeric rating scale, PACU: postoperative anesthesia care unit, POD: postoperative day; Non-TAP group: patients who did not undergo TAP block, TAP group: patients who underwent TAP block; *p*-value < 0.05 was considered statistically significant.

**Table 4:** Postoperative characteristics.

	All Patients	Non-TAP Group	TAP Group	*p*-Value
	(*n* = 142)	(*n* = 72)	(*n* = 70)	
Fentanyl at PACU (µg)	46.2 ± 28.4	48.3 ± 29.6	44.0 ± 27.2	0.575
Fentanyl at post-operation (µg)				
POD 1	65.0 ± 76.9	65.6 ± 79.3	64.3 ± 74.9	0.959
POD 3	148.4 ± 103.5	144.4 ± 115.4	152.5 ± 90.2	0.362
POD 5	169.1 ± 77.6	172.3 ± 78.8	165.7 ± 76.7	0.613
Time to pass flatus (POD)	2.8 ± 1.0	2.7 ± 0.9	2.9 ± 1.0	0.063
Hospital stay length (POD)	6.4 ± 1.6	6.6 ± 2.0	6.2 ± 1.2	0.297

All data are presented as the mean ± SD. PACU: postoperative anesthesia care unit, POD: postoperative day; Non-TAP group: patients who did not undergo TAP block, TAP group: patients who underwent TAP block; *p*-value < 0.05 was considered statistically significant.

**Table 5:** Postoperative complications.

	All Patients	Non-TAP Group	TAP Group	*p*-Value
	(*n* = 142)	(*n* = 72)	(*n* = 70)	
Nausea/vomiting	17 (12.0%)	9 (12.5%)	8 (11.4%)	0.844
Itching	16 (11.3%)	9 (12.5%)	7 (10.0%)	0.638
Anastomotic leak	2 (1.4%)	0 (0.0%)	2 (2.9%)	0.149
Anastomotic hemorrhage	2 (1.4%)	2 (2.8%)	0 (0.0%)	0.160
Infection of incision	2 (1.4%)	1 (1.4%)	1 (1.4%)	0.984
Ileus	2 (1.4%)	2 (2.8%)	0 (0.0%)	0.160

All data are presented as the number (%) of patients. Non-TAP group: patients who did not undergo TAP block, TAP group: patients who underwent TAP block; *p*-value < 0.05 was considered statistically significant.
